# Modeling Natural Anti-Inflammatory Compounds by Molecular Topology

**DOI:** 10.3390/ijms12129481

**Published:** 2011-12-20

**Authors:** María Galvez-Llompart, Riccardo Zanni, Ramón García-Domenech

**Affiliations:** 1 Molecular Connectivity & Drug Design Research Unit, Department of Physical Chemistry, Faculty of Pharmacy, University of Valencia, Avenida V.A. Estelles s/n, Burjasot, Valencia 46100, Spain; E-Mails: galloma@postal.uv.es (M.G.-L.); riccardo.zanni@studio.unibo.it (R.Z.); 2 Department of Pharmacology, Faculty of Pharmacy, University of Bologna, Via Irnerio, Bologna 48-40126, Italy

**Keywords:** Molecular Topology, virtual screening, natural, anti-inflammatory, linear discriminant analysis

## Abstract

One of the main pharmacological problems today in the treatment of chronic inflammation diseases consists of the fact that anti-inflammatory drugs usually exhibit side effects. The natural products offer a great hope in the identification of bioactive lead compounds and their development into drugs for treating inflammatory diseases. Computer-aided drug design has proved to be a very useful tool for discovering new drugs and, specifically, Molecular Topology has become a good technique for such a goal. A topological-mathematical model, obtained by linear discriminant analysis, has been developed for the search of new anti-inflammatory natural compounds. An external validation obtained with the remaining compounds (those not used in building up the model), has been carried out. Finally, a virtual screening on natural products was performed and 74 compounds showed actual anti-inflammatory activity. From them, 54 had been previously described as anti-inflammatory in the literature. This can be seen as a plus in the model validation and as a reinforcement of the role of Molecular Topology as an efficient tool for the discovery of new anti-inflammatory natural compounds.

## 1. Introduction

One of the biggest pharmacological problems today is the treatment of chronic inflammations. Diseases like chronic asthma, rheumatoid arthritis, multiple sclerosis, inflammatory bowel disease (IBD), and psoriasis, are strongly debilitating and are becoming increasingly common in our aging society. Rheumatoid arthritis and osteoarthritis are the major inflammatory diseases affecting people worldwide. Increases in life expectancy and aging populations are expected to make osteoarthritis the fourth leading cause of disability by the year 2020. Moreover, epidemiological studies have identified chronic infections and inflammation as major risk factors for various types of cancer [1].

Several classes of drugs, such as corticosteroids, NSAIDs, and biologics, are used to treat the inflammatory disorders. The main problem is that these drugs possess several adverse effects or are too expensive to be used. Corticosteroids have long been used for the management of rheumatoid arthritis and IBD’s diseases, but they suffer from some serious adverse effects, such as Cushing’s habitus, hypertension, hyperglycemia, muscular weakness, increased susceptibility to infection, osteoporosis, glaucoma, psychiatric disturbances, growth arrest, etc.

Likewise, the side effects associated with the use of NSAIDs, such as gastrointestinal ulceration and bleeding, and platelet dysfunction, are several and common, and because of the largest use (and abuse) of this class of drugs, they represent a big problem at the moment to treat chronic inflammations.

The coxibs also exhibited cardiovascular side effects due to inhibition of prostacyclin formation in the infarcted heart, tipping the balance of prostacyclin/thromboxane, coupled with a diminution in prostacyclin in heart muscle. Therefore, it is quite clear that the clinically used anti-inflammatory drugs suffer from the disadvantage of side effects and high cost of treatment (in case of biologics) [1].

There is a valid alternative to these drugs, represented by natural products, which offer a great hope in the identification of bioactive lead compounds and their development into drugs for treating inflammatory diseases [1]. Is known that plants have been the basis of many traditional medicine systems throughout the world for thousands of years and they represent an exhaustive source of “raw materials” in order to find and synthesize new molecules with pharmacological activity [1].

Natural Products (NP) are classified into three groups: NPs, semi-synthetic NPs or NP-derived [2]. The value of natural products can be assessed by the rate of introduction of new chemical entities of wide structural diversity, including serving as templates for semisynthetic and total synthetic modification.

An analysis of the origin of the drugs developed between 1981 and 2002 showed that natural products or natural product-derived drugs comprised 28% of all new chemical entities (NCEs) launched onto the market. In addition, 24% of these NCEs were synthetic or natural mimic compounds, based on the study of pharmacophores related to natural products. This combined percentage (52% of all NCEs) suggests that natural products are important sources for new drugs and are also good lead compounds suitable for further modification during the drug development process. Scrutiny of medical indications by source of compounds has demonstrated that natural products and related drugs are used to treat 87% of all categorized human diseases (48/55) [3].

It is noteworthy that Natural Products have played a pivotal role in immunosuppression drug discovery as shown by the launch of the NPs cyclosporin 72 (1983), tacrolimus (1993), sirolimus 10 (1999) and mycophenolate sodium (2003), and the semi-synthetic NPs mycophenolate mofetil (1995), everolimus 129 (2004) and fingolimod (2010). In addition, the NP-derived aspirin (acetylsalicylic acid) discovered in the late 1890s is still used widely as an analgesic and anti-inflammatory, while corticosteroids and b2 agonists modeled on adrenaline (e.g., salbutamol and salmeterol) are used to help control asthma [2]. A total of 13 NP and NP-derived drugs were approved for marketing worldwide from 2005 to 2007, with 5 being classified as NPs, 6 semi-synthetic NPs and 2 NP-derived drugs [2].

Despite this statistic, pharmaceutical companies have embraced the era of combinatorial chemistry, neglecting the development of natural products as potential drug candidates in favor of high-throughput synthesis of large compound libraries [4]. The main reasons for this include the incompatibility of natural product libraries with high-throughput screening and the marginal improvement in core technologies for natural product screening in the late 1980s and early 1990s [5]. Luckily, during the last years, the development of new technologies has revolutionized the screening of natural products. Applying these technologies compensates for the inherent limitations of natural products and offers a unique opportunity to re-establish natural products as a major source for drug discovery [5].

We have to understand that the natural product landscape offers, not only the direct introduction of natural products into the drug discovery process, but more often, natural products serve themselves as lead agents, providing the chemist with a structural platform which can be elaborated upon, or simplified, to yield a therapeutically valuable pharmaceutical [6]. They offer unmatched chemical diversity with structural complexity and biological potency. Natural product resources, especially from the marine environment, are resourceful and largely unexplored [7].

Another key point relating to natural products is that we can start from the original natural product and develop an analog strategy which permits us to create or modify new molecules with biological or pharmacological activity. As for the anti-inflammatory natural products, they have been discovered based on ethnopharmacological observations, thanks to some new strategies in chemical investigation. In this regard, through the use of topological descriptors, they could provide new potential drug targets.

In the late 1980s, computational chemistry sped up the drug discovery process. Afterwards, combinatorial chemistry (including molecular evolution, multiple parallel synthesis, *etc*.) arrived combined with High Throughput Screening (HTS), in the mid-1990s.

Virtual Screening, or *in silico* screening, is an approach attracting increasing interest in the pharmaceutical industry as a productive and cost-effective technology for the search of novel hit or lead compounds [8–10].

The principles involve the computational analysis of chemical databases, to identify those compounds that are most likely to show a given biological activity. Of course, these ideas are not new, but have been pursued for years by groups working in drug design and discovery. However, the availability of inexpensive high-performance computing platforms has transformed these processes in such a way that, at present, increasingly complex and more accurate analyses can be performed on a very large data set.

The topological virtual screening is based on the analysis of a chemical diversity of molecules [8], which enables the selection of the best potential molecular choices. In principle, the molecules are not classified according to their biological activity, but depicted by their topological indices (TIs) and after a computational study of their structures, only those ones complying with a desired topological model are chosen for further development.

Then a model comes from a linear discriminant analysis (LDA) containing two sets of structures: One of them has a well-defined pharmacological activity, and the other one, is built from structures showing no this biological activity.

The resulting model, associated with the desired pharmacological activity, generates a set of topological descriptors capable of differentiating potentially active compounds from those lacking activity.

The method above represents a rather detailed and relevant framework to search for leads, prioritizing the selection of compounds that are advisable to be tested in a biological assay. It offers a new option, a new method that shows itself to be powerful in facing the hunt for new targets, new lead compounds that finally enable the securing of new drugs.

This report deals with the search of natural anti-inflammatory compounds by using a database of natural products. The research team has gained experience in discovering new drugs applying Molecular Topology, and has developed several models in the field of anti-inflammatory compounds [11–13].

## 2. Materials and Methods

### 2.1. Analyzed Compounds

The model for searching new natural anti-inflammatory compounds was made up of 412 natural compounds, 123 active as anti-inflammatories and 289 inactive. Almost all the active compounds were from a paper reported by Kontogiorgis *et al.* [14] and the rest of them active and inactive from the collection Pure Natural Products from MicroSource database [15]. Compounds conforming the *test set* and Virtual screening were also achieved from these sources. Compounds forming the *training set* are shown in Supplementary material, Annex I. All sets of compounds are characterized by a large structural diversity

### 2.2. Molecular Descriptors

The 2D structure of each compound was drawn using the ChemDraw Ultra package [16]. Each compound was characterized by a set of 436 topological indices, standing among them the topological charge indices, quotients and differences between nonvalence and valence connectivity indices, topological and 2D autocorrelation descriptors. All indices were calculated with Dragon software [17]. In the supplementary material, Annex I, the TI’s values are given for all compounds of the model.

### 2.3. Modeling Techniques

Linear discriminant analysis (LDA) is a pattern recognition method which provides a classification model based on the combination of variables that best predict the category or group to which a given compound belongs. We built up a natural compounds database where all compounds were allocated into an active or inactive group according to their anti-inflammatory activity. The LDA was then applied to these two groups to obtain a discriminant function (*DF*) with the statistical software Statistica 9.0 [18]. The independent variables were the TIs, and the discriminatory property was the anti-inflammatory activity. The discriminant capability was assessed as the percentage of correct classifications in each set of compounds. The classification criterion was the minimal Mahalanobis distance (distance of each case to the mean of all the cases in a category). The quality of the discriminant function was evaluated using the Wilks parameter, λ, which was obtained by multivariate analysis of variance that tests the equality of group means for the variable in the discriminant model.

The method used to select the descriptors was based on the Fisher-Snedecor parameter (*F*), which determines the relative importance of candidate variables. The variables used to compute the linear classification function are chosen in a stepwise manner: at each step, the variable that makes the largest contribution to the separation of the groups is entered into the discriminant equation (or the variable that makes the smallest contribution is removed).

The validation of the selected function was done using an external *test set*. Compounds that comprise the *test set*, were randomly selected from approximately 20% of the data, and were not used in the set up of the *DF* equation.

Another important parameter that usually provides a balanced evaluation of the model’s prediction is the Matthews correlation coefficient (*MCC*) [19]. This coefficient is based on the fact that in any prediction process there can be four different possibilities to account for:

*TP* (True positive): Active compounds correctly classified or predicted.
*FP* (False positive): Inactive compounds classified as anti-inflammatory.
*TN* (True negative): Inactive compounds correctly classified.
*FN* (False negative): Anti-inflammatory compounds classified as inactive.

It is clear therefore, that any single number that represents the predictive power of the method must account for all the possibilities listed above. *MCC* fulfils these requirements. Matthews’ coefficient is defined as shown in Equation (1):

(1) $$MCC=\frac{\left(TP\times TN)-(FP\times FN\right)}{\sqrt{\left(TN+FN)\times (TN+FP)\times (TP+FP)\times (TP+FN\right)}}$$

The Matthews correlation coefficient ranges from −1 ≤ *MCC* ≤ 1. A value of *MCC* = 1 indicates the best possible prediction, in which every compound in the model was correctly classified, whereas if *MCC* = −1 then we are in the worst possible case (or anti-correlation), where no one single compound has been correctly labeled. Finally, a Matthews correlation coefficient of *MCC* = 0 is what would be expected for a random prediction.

### 2.4. Pharmacological-Activity Distribution Diagrams

A pharmacological distribution diagram (PDD) is a graphical representation that provides a straightforward way of visualizing the regions of minimum overlap between active and inactive compounds, as well as the regions in which the probability of finding active compounds is at a maximum [20].

Actually, a PDD is a frequency distribution diagram of dependent variables in which the ordinate represents the expectancy (probability of activity) and the abscissa represents the *DF* values in the range. For an arbitrary range of values of a given function, an “expectancy of activity” can be defined as Ea = a/(i + 1), where “a” is the number of active compounds in the range divided by the total number of active compounds and “i” is the number of inactive compounds in the interval divided by the total number of inactive compounds. The expectancy of inactivity is defined in a symmetrical way, as Ei = i/(a + 1). Presented with these diagrams, it is easy to visualize the intervals in which there is a maximum probability of finding new active compounds and a minimum probability of finding inactive compounds.

### 2.5. Topological Virtual Screening

The topological model resulting from *DF* function was used to find new natural anti-inflammatory compounds. A group of compounds from MicroSource Pure Natural Products Collection database, that has not been employed neither in the *training set* nor in the *test set*, were screened for the search of potential new anti-inflammatory natural compounds.

## 3. Results and Discussion

### 3.1. Similarity Study

A study of compounds’ similarity was previously carried out in order to guarantee that no simple or evident structural features are discriminating between the molecules that make up the data set. Thus, molecular weight, MW, partition coefficient, logP, (values estimated for log P with Dragon software, [17]) and Randic index, ^1^*χ*, have been calculated for all compounds in the database. These descriptors give us information about the molecular size, lipophilia and molecular branching, respectively.

**Figure 1:**
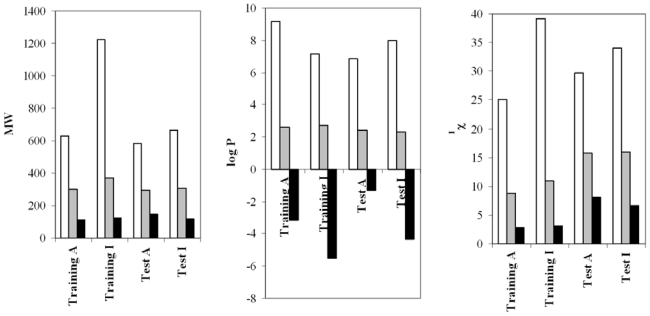
Average, maximum and minimum values (gray, white and black bars, respectively) obtained with molecular weight, MW, partition coefficient, logP, and Randic index, ^1^*χ* for the *training* and *test sets* of compounds.

For these parameters in the training set, we obtained an average value of 300 (MW), 2.66 (logP) and 10.4 (^1^*χ)* for the active compounds, whereas average values of 372 (MW), 2.7 (logP) and 10.9 (^1^*χ*) were obtained for the inactive ones. Hence, the set is well balanced and no obvious structural differences are expected to distort the study. The results obtained with the *test set* are similar to those of the *training set* (see Figure 1).

If we compare these values to those obtained for the selected set of anti-inflammatory natural compounds, *i.e.*, 276.75 (MW), 2.00 (logP) and 8.12 (^1^*χ*) value, we can see that the predicted anti-inflammatory natural compounds show lower values of the three parameters, and therefore the structures selected from natural compounds are diverse from those already well-known and used in the *training set*.

### 3.2. Mathematical Modeling

The mathematical model was developed from a *training set* including 412 compounds, with heterogeneous molecular structures. Even if the number of active compounds (123 molecules) and inactive (289) that comprise the *training set* were not similar in number, this was offset by the construction of a model by which every compound has the same statistical weight. The discriminant Equation (2), is shown below. This equation is comprised of five independent variables:

(2) $$\begin{array}{c} DF=-0,005\times \text{TI}1+5,666\times \text{ATS}7\text{m}-11,820\times \text{ATS}4\text{v}-\\ 9,178\times \text{ATS}7\text{v}+28,912\times \text{ATS}1\text{p}-42,202 \end{array}$$

N = 412, F = 46, and λ =0.663.

The molecular descriptors in Equation (2) are described in Table 1 along with their definitions and references.

According to Equation (2), a compound should be classified as active if *DF* > 0.78, otherwise it is labeled as inactive.

By applying this criterion to the *training set* (412 compounds), (see supplementary material annex I for details), 61 out of 123 experimentally active compounds were correctly classified as such (50% accuracy), and 284 out of 289 experimentally inactive compounds were also well classified (98% accuracy) as can be seen in Table 2. Altogether, the average of correct classification for the entire set of compounds (active plus inactive) was 74%.

The following formula was used to calculate the percentage of correctly classified compounds within a particular category (active or inactive) as shown in Equation (3):

(3) Classification accuracy (%)=(CCC×100/TNC)

where CCC is correctly-classified compounds and TNC is a total number of compounds.

Regarding the Matthews correlation coefficient, which returns a value between −1 and +1, our model shows a value of 0.6, what ensures its reliability.

Furthermore, the Matthews correlation coefficient was calculated in a slightly different way, *i.e.*, by adding +1 to each scale value, in this way the outcome it could be expressed as % accuracy. In other words, 0 would mean no correlation at all, 1 represents 50% and 2 stands for the maximum correlation (100%). By doing so, our model’s yield was 80% (*MCC* modified = 1.6).

To establish the adequate range of activity, we analyzed the pharmacological distribution diagram obtained with the discriminant function, *DF*.

**Figure 2:**
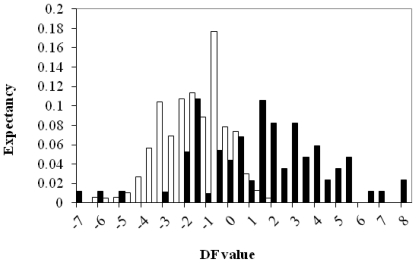
Pharmacological distribution diagram for natural anti-inflammatory compounds obtained using the discriminant function *DF*. (The black color represents the compounds with anti-inflammatory activity and the white color, the compounds without it).

Looking at Figure 2, we can appreciate that, all the compounds studied show *DF* values in the range 8 > *DF* > −7. Outside these ranges the compound’s classification is uncertain and it is labeled as “not-classified” (outliers), NC.

An easy way to evaluate the quality of the function above is to apply it into an external group. In our case, this group was made up of 84 compounds (41 active and 43 inactive) which had not been included for *DF* calculation, what is about 20% of the data. Table 3 outlines the results of the prediction obtained for every compound of the *test set.*

As we can appreciate in Table 2, the success rate is increased in the active group up to 59% (24 of 41 compounds analyzed were correctly classified). In the case of the inactive group belonging to the test set, there are only six compounds misclassified; the rate of correct compounds was 86% (37 of 43 compounds analyzed were correctly classified), indicating that *DF* has a high specificity in recognizing inactive compounds, because it has the capability to predict if an inactive compound is actually inactive. Hence, we can ensure that the number of “false active” is going to be minimumized. Furthermore, although *DF* will lead to the loss of some of the active compounds, the important point is that there is a lower risk of including false active compounds when we carry out a database screening searching for anti-inflammatory natural compounds.

As illustrated in Table 3, there is just one outlier or uncertain compound in the inactive group, namely Tropine, whose *DF* value exceeds the range of application of the model.

In Equation (2), there are topological descriptors which evaluate the molecular bonds, TI1, the atomic masses, ATS7m, Van der Waal volumes, ATS4v and ATS7v, and finally, the atomic polarizabilities of the molecules, ATS1p.

Although it is not easy to unfold the structural features explaining the discriminant equation obtained, some insight can be gained on the basis of the most relevant indices in the regression equation, namely TI1, ATS7m, ATS4v, ATS7v and ATS1p. Each one of these indices refers to a specific physical or chemical property of the molecule.

For example, the Moreau-Broto (ATS) autocorrelation descriptors represent the interactions between atoms at topological distance k, (lag k), for a particular atomic property (weighting factor). In our case, the weighting factors are basically the atomic polarizability, the van der Waals volume and the atomic mass. These descriptors seem to be sensitive to the molecular branching and cyclicity.

**Figure 3:**
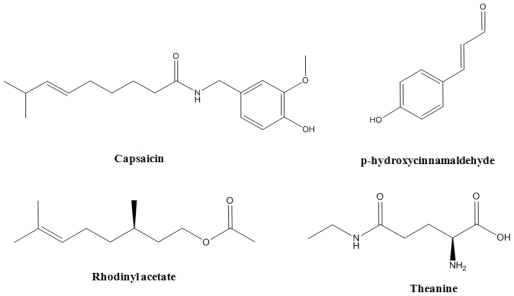
General overview of the active and inactive compounds by their structural and chemical properties.

From a general overview of the active and inactive compounds, we can find some differences, for example the active compounds typically show hydroxyl groups (low mass and electronic acceptors) which, contrary to the inactive compounds, are placed in the molecule far away from the carbonyls. On the other hand, the inactive set includes compounds showing methoxy groups (higher mass and electronic donors). In general there are less cyclic compounds among the inactive set. See as examples (Figure 3), capsaicin or p-hydroxycinnamaldehyde among the actives or Rhodinyl acetate or theanine among the inactives.

The active compounds often show a higher polarizability (taken into account by the index ATS1p), as compared to the inactive, which is compatible with their larger molecular volume and the presence of hydroxyl groups. Obviously the hydroxyl groups would also play a key role in molecular solubility and the molecule’s capability to form hydrogen bonding, which are also well known factors influencing the activity.

Given the high structural heterogenicity of the molecules used to build up the models, these results can be applied to large databases including natural compounds to search for new active compounds.

### 3.3. Topological Virtual Screening

Based on the model described above, a virtual screening was carried out on a database of heterogeneous natural compounds. We used some of the compounds of the library MicroSource Pure Natural Products Collection, that were not used for the construction of the model or for the external validation and we performed a virtual screening searching for anti-inflammatory natural compounds. The library composition can be obtained from the MicroSource Discovery Systems website [15].

As shown in Table 4, a set of 74 natural compounds were selected with a *DF* values between 2 < *DF* < 6 with predicted activity as anti-inflammatory. Almost all of these were commercially available.

As illustrated in the Table 4, most of the compounds selected had been described previously as anti-inflammatory in the literature (55/74) (see column 9), which is highly encouraging and represents an extra proof of the model’s performance. It is pretty clear that there are many ways of applying the model described herein to the search for new anti-inflammatory natural compounds. Although 19 out of the 74 compounds do not show anti-inflammatory activity, one cannot be sure if this is because of their inactivity or the absence of laboratory tests developed by someone. So it will be an attractive challenge for us to test these compounds and see if some of them would indeed show anti-inflammatory activity.

Virtual screening is increasingly gaining acceptance in the pharmaceutical industry as a cost-effective and timely strategy for analyzing very large chemical data set. This procedure is computationally intensive for analyzing large databases and it provides the most detailed basis for determining which compounds are likely to be potent hits or leads. The results outlined here demonstrate not only that the Topological Virtual Screening could accurately reproduce the well-known pharmacological activity, but also represent a new step forward in the pathway to demonstrate the high efficiency of the *in silico* methods based in Molecular Topology.

## 4. Conclusions

The joint use of topological-structural descriptors of compounds and a statistical treatment based on discriminant analysis has been demonstrated as a very efficient methodology for the selection of new natural compounds with anti-inflammatory activity. The mathematical model obtained can readily be applied to the search of new natural compounds in large databases or even for drug design. These results confirm the usefulness of Molecular Topology as a powerful tool in the search for new drugs.

## Supplementary Material



## Figures and Tables

**Table 1:** Descriptors used in this model.

Symbol	Name	Ref.
TI1	first Mohar index	[21]
ATS7m	2D Broto-Moreau autocorrelation of a topological structure-lag7/weighted by atomic masses	[22]
ATS4v	2D Broto-Moreau autocorrelation of a topological structure-lag4/weighted by Van der Waal volumes	[23]
ATS7v	2D Broto-Moreau autocorrelation of a topological structure-lag7/weighted by Van der Waal volumes	[23]
ATS1p	2D Broto-Moreau autocorrelation of a topological structure-lag4/weighted by Atomic polarizabilities	[23]

**Table 2:** Classification matrix obtained through the selected discriminant function (*DF*) for the *training* and *test set*.

	Percent-Correct	Compounds Classified as Active	Compounds Classified as Inactive
***Training Set***			

**Active Group**	50	61	62
**Inactive Group**	98	5	284

***Test Set***			

**Active Group**	59	24	17
**Inactive Group**	86	6	37

**Table 3:** Results of prediction of anti-inflammatory activity obtained applying the linear discriminant analysis to the *test set*.

Compound	TI1	ATS7m	ATS4v	ATS7v	ATS1p	*DF*	Class.	Ref.

						Value	Pred.	
**Active Group**								

2′,β-DIHYDROXYCHALCONE	25.6	2.79	2.81	2.57	2.91	0.82	A	[24]
3,7-DIMETHOXYFLAVONE	49	3	3.27	2.73	3.01	−2.19	I	[25]
4-METHYLESCULETIN	11.21	1.02	2.51	0.23	2.59	6.44	A	[26]
ANISODAMINE	66.85	3.2	3.02	2.93	3.05	1.22	A	[27]
BERGENIN	51.11	3.32	3.37	2.52	2.97	−0.82	I	[28,29]
β-CARYOPHYLLENE ALCOHOL	23.75	0	3.12	0	2.92	5.13	A	[30]
BICUCULLINE (+)	158.89	3.69	3.66	3.24	3.25	−0.95	I	[31,32]
BOLDINE	73.83	3.38	3.7	2.83	3.16	−1.75	I	[33,34]
BRAZILEIN	58.35	2.98	3.35	2.24	3.08	3.31	A	[35,36]
CAMPTOTHECIN	113.6	3.45	3.64	3.14	3.27	−0.33	I	[37,38]
CAPSAICIN	0	3.01	2.95	2.71	3	1.8	A	[39,40]
CARNOSIC ACID	54.11	2.93	3.81	2.68	3.21	−2.66	I	[41]
CARVACROL	0	0	2.08	0	2.44	3.68	A	[14]
DIHYDROTANSHINONE I	57.02	2.66	3.51	2.4	3.13	−0.49	I	[42,43]
ELLAGIC ACID	56.29	2.61	3.43	1.72	3.02	3.35	A	[44,45]
EPIAFZELECHIN (2*R*,3*R*)(−)	44.79	3.02	3.06	2.53	2.98	1.45	A	[46]
EUPHOL	146.22	3.66	4.11	3.61	3.54	−1.62	I	[47]
GALLIC ACID	0	0	1.93	0	2.33	2.36	A	[48]
GENISTEIN	44.57	2.88	3.09	2.45	2.98	1.15	A	[14]
HARMALINE	26.93	1.98	2.93	1.48	2.8	1.62	A	[49]
HEMATEIN	63.65	3.11	3.4	2.34	3.1	3.01	A	[50,51]
HIERACIN	53.56	3.3	3.17	2.72	3.03	1.38	A	[52]
IRIGENIN TRIMETHYL ETHER	90.88	3.73	3.75	3.27	3.17	−4.15	I	[53]
ISOLIQUIRITIGENIN	29.12	2.87	2.78	2.61	2.94	2	A	[54]
KOPARIN 2′-METHYL ETHER	59.21	3.2	3.37	2.81	3.05	−1.78	I	[55]
KYNURENINE	0	2.45	2.51	1.7	2.61	1.89	A	[56]
LAWSONE	9.48	0	2.43	0	2.59	4	A	[57]
LUTEOLIN GLUCOSIDE	171.97	4.01	3.57	3.45	3.35	2.44	A	[14]
MADECASSIC ACID	189.69	4.04	4.43	3.75	3.63	−2.1	I	[58]
METHYL ORSELLINATE	0	0.85	2.39	0.41	2.42	0.59	I	[59,60]
*N*-METHYLANTHRANILIC ACID	0	0	2.14	0	2.32	−0.42	I	[61]
OLEANOLIC ACID	163.48	3.85	4.28	3.68	3.59	−1.63	I	[14]
OUABAIN	358.36	4.4	4.26	3.88	3.66	0.84	A	[62]
p-HYDROXYCINNAMALDEHYDE	0	1.3	1.95	0.71	2.39	4.75	A	[63,64]
QUERCETIN	52.38	3.27	3.2	2.67	3.03	1.29	A	[65]
QUERCETIN TETRAMETHYL (5,7,3′,4′) ETHER	74.79	3.59	3.54	3.09	3.11	−2.49	I	[14]
SANTONIN	31.04	1.86	3.2	1.19	2.94	4.27	A	[66]
SILIBININ	243.99	4.1	3.85	3.61	3.48	1.68	A	[67–69]
TECTORIGENIN	53.39	3.12	3.26	2.68	3.03	−0.42	I	[70,71]
UMBELLIFERONE	8.36	0	2.12	0	2.47	4.15	A	[14]
UVAOL	153.27	3.74	4.29	3.61	3.58	−2.13	I	[72]

**Inactive Group**								

2-METHYL GRAMINE	13.23	1.1	2.73	1.1	2.65	−1.78	I	-
3-DEACETYLKHIVORIN	285.72	4.57	4.44	4.14	3.62	−3.59	I	-
3-PINANONE OXIME	9.02	0	2.19	0	2.56	5.84	A	-
ASARYLALDEHYDE	0	1.2	2.61	0.92	2.41	−4.9	I	-
BAEOMYCESIC ACID	47.58	3.65	3.52	3.25	3.16	−1.82	I	-
BOVINOCIDIN (3-NITROPROPIONIC ACID)	−8.58	0	1.01	0	1.63	−6.92	I	-
CHOLEST-5-EN-3-ONE	128.51	3.42	3.89	3.37	3.45	−0.65	I	-
CONESSINE	112.88	3.25	3.84	3.16	3.36	−1.57	I	-
CRASSIN ACETATE	37.49	3.91	3.71	3.52	3.23	−3.06	I	-
CRINAMINE	78.42	2.9	3.48	2.38	3.09	0.22	I	-
DEACETOXY-7-OXISOGEDUNIN	188.23	3.82	4.25	3.46	3.5	−2.23	I	-
DEOXYANDIROBIN	161.25	3.95	4.14	3.62	3.47	−2.48	I	-
DIPHENYLUREA	21.73	2.4	2.5	2.4	2.77	−0.23	I	-
DUARTIN. DIMETHYL ETHER	76.8	3.39	3.6	3.04	3.11	−3.9	I	-
EPI(13)TORULOSOL	30.87	3.08	3.56	2.87	3.13	−2.72	I	-
EUDESMIC ACID	0	1.3	2.7	0.71	2.45	−2.3	I	-
EVOXINE	76.98	3.47	3.43	2.99	3.08	−1.8	I	-
GLUCITOL-4-GUCOPYANOSIDE	0	3.66	3.06	2.82	2.83	−1.71	I	-
HEXAMETHYLQUERCETAGETIN	88.94	3.82	3.75	3.33	3.17	−4.27	I	-
ISOOSAJIN	153.22	3.79	3.85	3.61	3.42	−1.22	I	-
JUAREZIC ACID	0	2.23	2.2	1.8	2.56	1.88	A	-
KHAYASIN	274.26	4.48	4.38	4.11	3.65	−2.18	I	-
LEOIDIN	68.47	3.83	3.74	3.25	3.22	−1.74	I	-
LOMATIN	31.81	2.35	3.04	1.99	2.88	−0.07	I	-
MEDICARPIN	56.7	2.78	3.17	2.43	3.01	0.37	I	-
MEROGEDUNIN	72.24	3.21	3.94	2.75	3.27	−1.63	I	-
METAMECONINE	12.34	0.85	2.5	0.41	2.5	1.53	A	-
METHYL EVERNINATE	0	1.61	2.56	0.93	2.46	−0.68	I	-
METHYL ROBUSTONE	146.49	3.65	3.68	3.34	3.3	−1.03	I	-
*N*-METHYLISOLEUCINE	−11.17	0	2.05	0	2.1	−5.69	I	-
PLECTOCOMINE METHYL ETHER	23.69	1.52	2.76	1.21	2.74	1.61	A	-
PODOTOTARIN	346.77	4.54	4.56	4.38	3.85	−1.06	I	-
PRENYLETIN	24.04	2.78	2.76	2.43	2.82	−0.07	I	-
PTAEROXYLIN	35.11	2.49	3.15	2.26	2.93	−1.55	I	-
RETUSOQUINONE	0	1.3	2.35	0.71	2.52	3.8	A	-
RHETSININE	94.88	3.23	3.46	2.98	3.19	−0.36	I	-
RHODINYL ACETATE	−25.56	2.04	2.36	1.8	2.52	−2.14	I	-
ROBUSTIC ACID	114.56	3.69	3.71	3.36	3.28	−1.77	I	-
SARMENTOSIDE B	484.87	4.66	4.42	4.15	3.75	−0.04	I	-
SENECRASSIDIOL 6-ACETATE	41.44	2.6	3.42	2.06	3.04	0.77	I	-
THEANINE	−17.76	1.76	2.01	1.31	2.22	−3.61	I	-
TROPINE	5.68	0	1.22	0	2.34	10.88	N.C.	-
XANTHOXYLIN	0	1.2	2.63	0.92	2.46	−3.75	I	-

**Table 4:** Group of natural compounds selected as anti-inflammatories. The indexes value for each compounds, the *DF* value and the known anti-inflammatory activity are outlined.

Compound	TI1	ATS7m	ATS4v	ATS7v	ATS1p	*DF* Value	Ref.
1,5-NORCARYOPHYLLEN-3-ONE	12.95	0	3.07	0	2.8	2.43	[73]
2′,4′-DIHYDROXYCHALCONE 4′-GLUCOSIDE	119.2	3.71	3.36	3.29	3.28	3.08	nr
2-HYDROXYXANTHONE	24.98	1.73	2.85	1.39	2.82	2.58	[74]
3-HYDROXYCOUMARIN	8.25	0	2.15	0	2.47	3.79	[75]
3-NOR-3-OXOPANASINSAN-6-OL	24.04	0	3.03	0	2.89	5.32	nr
alpha-TOCHOPHEROL	83.77	3.37	3.68	3.28	3.45	2.53	nr
APIIN	345.29	4.46	3.8	3.88	3.55	3.35	[76]
AVOCADANE ACETATE	−70.27	3.07	2.96	2.76	3.01	2.16	nr
BATYL ALCOHOL	−83.55	2.94	2.96	2.81	3.08	3.3	[77]
BERGAPTOL	22.16	1.3	2.67	0.71	2.69	4.85	[78]
BIXIN	−103.7	3.31	3.33	3.18	3.27	2.98	[79]
BRAZILIN	58.35	2.98	3.35	2.24	3.08	3.31	[80]
CANTHARIDIN	18.37	0	2.32	0	2.62	6.04	nr
CAPSANTHIN	187.67	3.83	3.96	3.77	3.77	6.06	[81]
CARYOPHYLLENE [t(−)]	11.37	0	2.89	0	2.77	3.75	[82]
CEDROL	23.5	0	3.14	0	2.92	4.87	[83]
CHAULMOOGRIC ACID	0	2.69	2.77	2.57	2.99	3.14	[84]
CHLOROGENIC ACID	48.22	3.36	3.09	2.78	3.07	3.29	[85]
CINEOLE	6.55	0	2.08	0	2.48	4.81	[86]
COSMOSIIN	162.44	3.92	3.54	3.41	3.33	2.27	[87]
COTININE	11.53	0.85	2.43	0.41	2.53	3.21	[88]
CULMORIN	25.72	0	3.2	0	2.94	4.8	nr
DESOXYPEGANINE HYDROCHLORIDE	16.37	0	2.49	0	2.65	4.82	nr
DIALLYL SULFIDE	−7.5	0	1.39	0	2.12	2.63	[89]
DICTAMNINE	22.62	0	2.88	0	2.71	2.05	nr
DIGITOXIN	1046.2	4.61	4.4	4.15	3.93	2.34	[62]
DIGOXIN	1069.1	4.64	4.43	4.16	3.94	2.11	[62]
DJENKOLIC ACID	−28.53	2.37	2.28	1.86	2.69	4.92	[90]
EPOXY (1,11)HUMULENE	15.16	0	3.05	0	2.83	3.48	[91]
GARLICIN	−9.74	0.69	1.64	0.69	2.4	5.34	[92]
GIBBERELLIC ACID	92.61	2.83	3.8	2.26	3.27	2.08	[93]
GUAIAZULENE	13.61	0	3	0	2.83	4.22	[94]
GUVACINE HYDROCHLORIDE	0	0	1.32	0	2.1	2.91	nr
HAEMATOXYLIN	63.65	3.11	3.4	2.34	3.1	3.01	nr
HARMALOL HYDROCHLORIDE	22.47	1.65	2.81	0.87	2.77	5.94	nr
HARMANE	19.16	0	2.76	0	2.74	4.34	[95]
HARMOL HYDROCHLORIDE	22.47	1.65	2.81	0.87	2.77	5.94	[96]
HARPAGOSIDE	218.6	4.16	3.56	3.65	3.4	2.86	[97]
HELENINE	28.19	1.79	3.07	1.26	2.91	4.05	[98]
HESPERIDIN	421.88	4.48	3.87	3.88	3.6	3.8	[99]
HINOKITIOL	0	0	2.26	0	2.48	2.74	[100]
HUMULENE (alpha)	0	0	3	0	2.77	2.56	[82]
INDOLE-3-CARBINOL	7.49	0	2.09	0	2.46	4.19	[101]
INOSITOL	0	0	1.73	0	2.28	3.12	[102]
ISOBERGAPTENE	25.9	0.85	2.91	0.41	2.72	2.91	nr
ISOKOBUSONE	14.57	1.3	3.12	0.71	2.83	3.55	nr
JUGLONE	9.48	0	2.47	0	2.59	3.48	[103]
KOBUSONE	26.62	0	3.12	0	2.85	3.35	nr
L(+/−)-ALLIIN	−14.57	1.3	1.97	0.71	2.36	3.65	[104]
LYCOPODINE PERCHLORATE	36.79	0	3.48	0	3.01	3.55	[105]
MENADIONE	9.48	0	2.53	0	2.63	3.95	[106]
MENTHOL(−)	0	0	2.2	0	2.44	2.26	[107]
MENTHONE	0	0	2.2	0	2.44	2.26	[108]
MIMOSINE	0	2.31	2.25	1.23	2.43	3.19	[109]
MUUROLLADIE-3-ONE	15	1.3	3.09	0.71	2.86	4.75	nr
OCTOPAMINE HYDROCHLORIDE	0	0.94	1.94	0.3	2.36	5.54	nr
PATULIN	7.06	0	1.81	0	2.28	2.2	[110]
PECTOLINARIN	427.48	4.54	3.93	3.96	3.61	3.01	[111]
PHLORIDZIN	127.51	4.08	3.47	3.57	3.31	2.28	[112]
PICROTOXININ	68.36	2.09	3.46	1.2	3.05	5.58	nr
PIPERINE	74.81	2.87	2.93	2.6	3	2.01	[113]
PLUMBAGIN	10.92	0	2.67	0	2.67	3.28	[114]
PUNCTAPORONIN B	17.9	1.41	3.3	0.57	2.91	5.6	nr
PURPURIN	33.76	2.12	3.22	1.62	2.96	2.28	[115]
RHAPONTIN	125.6	3.82	3.42	3.38	3.28	2.06	[116]
RHOIFOLIN	357.18	4.51	3.85	3.93	3.57	3.3	[117]
RUTOSIDE (rutin)	392.19	4.68	3.88	4.07	3.6	3.12	[118]
SAFROLGLYCOL	14.58	2.26	2.4	1.4	2.54	2.79	[119]
SCOPOLETIN	11.82	1.33	2.47	0.82	2.54	2.11	[120]
SECURININE	31.65	0.85	3.02	0.41	2.85	5.28	nr
SHIKIMIC ACID	0	0	1.93	0	2.33	2.36	[121,122]
THYMOQUINONE	0	0	2.31	0	2.48	2.11	[123]
TRYPTAMINE	9.75	0.77	2.38	0.53	2.56	3.05	[124]
XANTHURENIC ACID	13.51	1.3	2.65	0.71	2.64	3.73	[56]

nr (is not referenced as an anti-inflammatory in literature).
