# Supplementary material for: Modeling Natural Anti-Inflammatory Compounds by Molecular Topology
Source: Int J Mol Sci. 2011 Dec 20;12(12):9481–503. doi: 10.3390/ijms12129481 (PMC3257142; doi:10.3390/ijms12129481)
Supplement: Supplementary file 1 [file ijms-12-09481-s001.pdf]

Supplementary Material

Annex 1. Compounds used in the training set and results obtained applying the linear discriminant analysis (Eq. 1) to anti-inflammatory natural compounds.

COMPOUND	TI1	ATS7m	ATS4v	ATS7v	ATS1p	DF value	Predicted activity
**Active Group**							
2',3-DIHYDROXY-4,4',6'-TRIMETHOXYCHALCONE	43.43	3.32	3.28	2.94	3.05	−1.1	I
2',4-DIHYDROXY-3,4',6'-TRIMETHOXYCHALCONE	43.34	3.32	3.28	2.94	3.05	−1.1	I
2-ACETYLPYRROLE	0	0	1.39	0	2.04	0.38	I
2-HYDROXY-3,4-DIMETHOXY BENZOIC ACID	0	1.3	2.52	0.71	2.41	−1.29	I
4-METHOXY-4'-HYDROXY-DALBERGIONE	26.84	2.94	3.23	2.45	2.96	−0.82	I
4-METHOXYDALBERGIONE	24.37	2.71	3.21	2.31	2.94	−1.3	I
4'-METHOXYFLAVONE	33.69	3.11	3.11	2.81	2.98	−1.08	I
ABRINE (L)	19.07	2.35	2.76	1.91	2.74	−0.06	I
ACACETIN	50.82	3.03	3.15	2.65	3.01	0.02	I
ALLICIN	−11.05	0.69	1.83	0.69	2.47	5.12	A
AMYGDALIN	135.88	4.07	3.45	3.47	3.28	2.27	A
ANETHOLE	0	1.2	2.14	0.92	2.39	−0.04	I
APIGENIN	44.89	2.91	3.07	2.53	2.98	0.8	A
ARTENIMOL	45.24	1.2	3.38	0.92	2.97	1.77	A
ARZANOL	52.34	3.88	3.68	3.49	3.28	−1.08	I
ASARININ (−)	173.69	3.52	3.45	3.12	3.24	1.1	A
ASTAXANTHIN	191	3.85	4.05	3.74	3.78	5.8	A
AUSTRICINE	34.29	1.69	3.38	0.83	2.96	5.2	A
AZELAIC ACID	−23.92	2.12	2.09	1.62	2.38	−0.74	I
BENZYL ISOTHIOCYANATE	0	1.3	1.87	0.74	2.39	5.4	A
BERGAPTEN	26	1.61	2.91	0.93	2.72	2.48	A
BIOCHANIN A	50.47	2.99	3.18	2.61	3.01	−0.11	I
BISABOLOL	0	2.2	2.74	2.2	2.8	−1.36	I
CADINENE	13.39	0	3.05	0	2.83	3.65	A
CAFFEIC ACID	0	2.15	2.15	1.12	2.47	5.73	A
CARMINIC ACID	185.61	4.31	3.93	3.6	3.44	1.26	A
CARNOSOL	72.73	2.85	3.78	2.64	3.23	−1.93	I
CARYOPHYLLENE	12.95	0	3.14	0	2.83	2.59	A
CELASTROL	167.15	3.9	4.23	3.66	3.58	−1.03	I
CEPHARANTHINE	443.28	4.81	4.34	4.54	3.79	−0.61	I
CHAMAZULENE	11.72	0	2.89	0	2.77	3.75	A
CHICORIC ACID	94.06	3.93	3.48	3.35	3.35	4.46	A
CHLORO~1	48.22	3.36	3.09	2.78	3.07	3.29	A
CHRYSIN	40.1	2.75	3.04	2.45	2.96	0.33	I
CHRYSOPHANOL	34.07	2.04	3.27	1.8	2.99	0.43	I
CITRININ	17.98	1.99	3.17	1.4	2.82	0.2	I
CORTISONE	89.09	3.39	3.83	2.95	3.31	−0.21	I
COUMARIN	6.89	0	2.02	0	2.43	4.14	A
CROCETIN	−70.98	3.25	3.05	2.95	3.08	2.59	A
CRUSTECDYSONE	171.69	3.85	4.09	3.55	3.53	−0.08	I
CURCUMIN	65.37	3.46	3.27	3.03	3.2	3.25	A
CYANIDIN	49.15	3.14	3.11	2.61	3.01	1.51	A
CYTISINE	18.37	0	2.69	0	2.68	3.41	A
DAPHNETIN	9.68	0	2.29	0	2.51	3.2	A
DHA	−84.25	2.93	3	2.84	3.13	3.91	A
DIMETHYLCAFFEIC ACID	0	2.36	2.61	1.62	2.54	−1.02	I
DIOSGENIN	175.97	3.59	3.99	3.42	3.51	0.05	I
EMODIN	37.7	2.34	3.32	1.95	3.01	0.73	I
ENOXOLONE	173.87	4.02	4.3	3.75	3.61	−1.28	I
EPA	−70.79	2.81	2.89	2.71	3.04	2.93	A
ERGOSTA-7,22-DIEN-3-ONE	138.78	3.55	3.91	3.5	3.48	−0.53	I

Annex 1. *Cont.*

ERGOSTEROL	138.78	3.55	3.91	3.5	3.48	−0.53	I
ERYTHRODIOL	154.1	3.76	4.25	3.64	3.58	−1.76	I
ESCULETIN	9.8	1.02	2.23	0.23	2.51	7.47	A
ESCULIN	69.39	3.48	3.14	2.89	3.02	0.84	A
EUGENOL	0	1.2	2.31	0.92	2.43	−0.85	I
FARNESOL	−32.85	2.34	2.64	2.25	2.74	−1.54	I
FORMONONETIN	46.73	2.85	3.1	2.53	2.98	0.08	I
FRAXETIN	13.17	1.33	2.61	0.82	2.58	1.45	A
GALACTURONICACID	−17.66	1.52	2.07	0.42	2.22	2.28	A
GAMBOGIC ACID	443.87	4.8	4.42	4.5	3.83	−0.08	I
GENTISIC ACID	0	0	1.81	0	2.28	2.45	A
GOSSYPOL	192.31	4.2	4.33	3.86	3.63	−1.02	I
HAEMATOMMIC ACID	0	0	2.52	0	2.51	0.47	I
HECOGENIN	184.19	3.7	4.04	3.47	3.52	−0.02	I
HERNIARIN	10.39	1.2	2.33	0.92	2.51	1.02	A
HESPERETIN	55.06	3.22	3.22	2.73	3.03	0.19	I
HOMOVANILLICACID	0	1.98	2.31	0.94	2.42	3.11	A
HYDROXYTYROSOL	0	1.02	1.88	0.23	2.34	6.81	A
HYPERICIN	256.55	4.57	4.57	4.05	3.73	−1.04	I
IMPERATORIN	50.32	3.01	3.09	2.68	2.95	−1.07	I
IRIDIN	217.19	4.28	3.86	3.72	3.42	0.17	I
ISOGINKGETIN	416.72	4.49	4.14	4.11	3.7	1.39	A
ISOPEONOL	0	0	2.31	0	2.38	−0.68	I
KAEMPFEROL	47.91	3.09	3.16	2.57	3.01	1	A
KOJIC ACID	0	0	1.46	0	2.11	1.62	A
KOPARIN	54.36	3.12	3.24	2.68	3.03	−0.18	I
LEUCODIN	31.32	1.69	3.28	0.83	2.94	5.71	A
LIMONENE	0	0	1.95	0	2.4	4.13	A
LINALOOL (+)	−14.9	1.1	2.02	1.1	2.35	−1.96	I
LINALYL ACETATE	−21.95	1.99	2.64	1.87	2.52	−6.52	I
LINAMARIN	0	2.56	2.67	1.76	2.59	−0.65	I
LINOLEICACID	−58.5	2.69	2.77	2.57	2.94	1.96	A
LUPININE	8.62	0	2.43	0	2.51	1.64	A
LUTEOLIN	49.29	3.13	3.11	2.63	3.01	1.23	A
LYCOPENE	−195.8	3.66	3.76	3.66	3.69	8.11	A
MANDELIC ACID. METHYL ESTER	0	0.69	2.15	0.69	2.38	−1.21	I
NARINGIN	357.18	4.51	3.85	3.93	3.57	3.3	A
NOBILETIN	89.53	3.67	3.76	3.27	3.17	−4.65	I
NORDIHYDROGUARETIC ACID	38.91	3.16	3.09	2.84	3.08	2.04	A
OLEUROPEIN	193.64	4.18	3.73	3.72	3.44	1.78	A
ORSELLINIC ACID	0	0	2.19	0	2.38	0.8	A
PARTHENOLIDE	32.76	1.79	3.24	1.26	2.91	1.85	A
PEONIFLORIN	244.22	4.44	3.62	3.88	3.41	1.87	A
PHENETHYLISOTHIOCYANATE	0	1.99	2.01	1.43	2.48	3.78	A
PHLOROACETOPHENONE	0	0	2.19	0	2.38	0.81	A
PICROCROCIN	34.58	3.4	3.21	3	3	−1.87	I
PIMPINELLIN	32.7	1.63	3.18	1.02	2.78	0.29	I
PINENE	5.89	0	1.79	0	2.49	8.43	A
PRISTIMERIN	180.76	4	4.27	3.78	3.59	−1.79	I
PROANTHOCYANIDINS/E	402.08	4.7	4.21	4.26	3.71	0.79	A
PSORALEN	19.55	1.3	2.51	0.71	2.66	5.77	A
PTEROSTILBENE	28.6	2.87	2.88	2.61	2.91	−0.06	I
PYRIDOXINE	0	0	2.19	0	2.36	0.26	I
RESERPINE	456.56	4.47	4.23	4.07	3.67	−0.22	I
RESVERATROL	23.12	2.75	2.63	2.45	2.85	2.29	A
RHAMNOSE	−13.25	0	1.85	0	2.11	−2.89	I
ROSMARINIC ACID	55.96	3.6	3.14	2.99	3.14	4.03	A
RUTIN	392.19	4.68	3.88	4.07	3.6	3.12	A
SAFRANAL	0	0	2.26	0	2.44	1.61	A
SAFROLE	9.9	1.2	2.26	0.92	2.47	0.88	A
SPATHULENOL	25.7	0	3.2	0	2.92	4.12	A

Annex 1. *Cont.*

S-PROPENYLCYSTEINE	−14.41	1.3	1.92	0.71	2.38	4.96	A
SQUALENE	−112	3.3	3.43	3.3	3.4	4.52	A
STIGMASTEROL	150.8	3.6	3.97	3.56	3.51	−0.64	I
STROPHANTHIDIN	141.2	3.61	3.96	3.22	3.41	−0.2	I
SYRINGIC	0	1.47	2.63	1.26	2.45	−5.57	I
THYMOL	0	0	2.2	0	2.44	2.26	A
TRIGONELLINE	0	0	1.75	0	2.17	−0.03	I
TRYPTANTHRIN	46.99	2.23	3.22	2.14	3	−0.89	I
URSOLIC ACID	162.65	3.83	4.32	3.65	3.59	−1.99	I
USNIC ACID	59.31	3.43	3.7	2.75	3.16	−0.6	I
VULPINIC ACID	70.78	3.28	3.45	3	3.14	−1.4	I
**Inactive Group**							
1(2)alpha-EPOXYDEOXY DIHYDROGEDUNIN	233.41	4.3	4.33	3.9	3.56	−3.18	I
1,2alpha-EPOXY-7-DEACETOXY-7-OXODIHYDROGEDUNIN	231.31	3.91	4.27	3.5	3.53	−1.89	I
1,3-DIDEACETYLDEOXYKHIVORIN	192.05	4.3	4.33	3.9	3.54	−3.31	I
1,4,5,8-TETRAHYDROXY-2,6-DIMETHYLANTHROQUINONE	44.2	2.92	3.48	2.36	3.08	0.4	I
1,7-DIDEACETOXY-1,7-DIOXOKHIVORIN	246.72	4.09	4.36	3.7	3.57	−2.51	I
10-HYDROXYCAMPTOTHECIN	123.4	3.54	3.66	3.17	3.29	−0.03	I
11alpha-ACETOXYKHIVORIN	385.91	5.05	4.63	4.55	3.73	−4.13	I
11-OXOURSOLIC ACID ACETATE	209.54	4.14	4.45	3.85	3.66	−1.98	I
12a-HYDROXY-5-DEOXYDE HYDROMUNDUSERONE	87.88	3.43	3.52	2.99	3.14	−1.58	I
12a-HYDROXY-9-DEMETHYLMUNDUSERONE 8-CARBOXYLIC ACID	104.89	3.75	3.71	3.1	3.22	−0.85	I
1-METHYLXANTHINE	8.29	0	2.07	0	2.26	−1.5	I
1R,9S-HYDRASTINE	147.23	3.72	3.73	3.28	3.25	−1.99	I
2,3-DIHYDROISOGEDUNIN	229.28	4.29	4.34	3.89	3.56	−3.29	I
2,3-DIHYDROXY-4-METHOXY-4'-ETHOXYBENZOPHENONE	31.28	3	3.14	2.53	2.95	−0.38	I
2,3-METHANO-7,2'-DIMETHOXYFLAVANONE	76.1	2.91	3.42	2.71	3.1	−1.85	I
2,5-DIHYDROXY-3,4-DIMETHOXY-4'-ETHOXYBENZOPHENONE	35.93	3.21	3.33	2.75	3	−2.09	I
2'-METHOXYFORMONETIN	55.35	3.09	3.29	2.75	3.03	−1.6	I
2-METHOXYRESORCINOL	0	0	1.76	0	2.18	−0.08	I
2-METHYLENE-5-(2,5-DIOXO)	53.08	3.03	3.52	2.65	3.1	−1.51	I
3,4',5,6,7-PENTAMETHOXYFLAVONE	77.87	3.6	3.65	3.16	3.13	−3.86	I
3,4,5-TRIMETHOXYCINNAMALDEHYDE	0	2.32	2.86	1.71	2.58	−3.94	I
3',4'-DIMETHOXYFLAVONE	51.89	3.08	3.22	2.73	3.01	−1.19	I
3,5-DIHYDROXYFLAVONE	39.24	2.57	3.09	2.36	2.96	−0.43	I
3beta-ACETOXYDEOXODIHYDROGEDUNIN	279.33	4.42	4.42	4.02	3.61	−3.37	I
3beta-ACETOXYDEOXYANGOLENSIC ACID METHYL ESTER	223.48	4.27	4.35	3.88	3.55	−3.48	I
3-DEOXO-3beta-HYDROXY MEXICANOLIDE 16-ENOL ETHER	204.13	4.17	4.26	3.77	3.53	−2.58	I
3-HYDROXYFLAVONE	35.68	2.34	3.02	2.25	2.94	−0.61	I
3-METHOXYCATECHOL	0	0	1.67	0	2.18	1.01	A
3-METHYLORSELLINIC ACID	0	0	2.39	0	2.47	0.97	A
4,4'-DIMETHOXYDALBERGIONE	29.89	3.04	3.31	2.61	2.98	−1.97	I
4-HYDROXY-6-METHYLPYRAN-2-ONE	0	0	1.4	0	2.06	0.68	I
4-O-METHYLPHLORACETOPHENONE	0	1.2	2.39	0.92	2.42	−2.03	I
5,2'-DIMETHOXYFLAVONE	50.43	3	3.3	2.71	3.01	−2.53	I
5,7-DIHYDROXYISOFLAVONE	39.82	2.57	3.07	2.36	2.96	−0.16	I
5,7-DIMETHOXYISOFLAVONE	49.48	2.85	3.27	2.64	3.01	−2.36	I

Annex 1. *Cont.*

6,3'-DIMETHOXYFLAVONE	52.15	2.95	3.19	2.66	3.01	−1.04	I
6,4'-DIMETHOXYFLAVONE	52.49	2.87	3.17	2.61	3.01	−0.75	I
6-ACETOXYANGOLENSIC ACID METHYL ESTER	234.38	4.31	4.39	3.86	3.57	−3.13	I
6-HYDROXYANGOLENSIC	195.65	4.06	4.31	3.65	3.51	−3.08	I
7,2'-DIMETHOXYFLAVONE	51.1	3.11	3.23	2.81	3.01	−1.98	I
7-DEACETYLKHIVORIN	291.13	4.42	4.44	4.02	3.62	−3.29	I
7-DESACETOXY-6,7-DEHYDROGEDUNIN	181.1	3.76	4.19	3.42	3.49	−1.97	I
7-OXOCALLITRISIC ACID, METHYL ESTER	57.49	3.03	3.76	2.81	3.21	−2.72	I
7-OXOCHOLESTEROL	134.1	3.51	3.94	3.4	3.46	−0.72	I
7-OXOCHOLESTERYL ACETATE	165.35	3.78	4.04	3.61	3.52	−0.7	I
8,2'-DIMETHOXYFLAVONE	50.36	3.13	3.28	2.79	3.01	−2.31	I
8beta-HYDROXYCARAPIN 3,8-HEMIACETAL	230.38	4.16	4.33	3.69	3.54	−2.41	I
8-HYDROXY-15,16-BISNOR-11-LABDEN-13-ONE	24.02	2.4	3.51	2.14	3.04	−2.04	I
8-HYDROXYCARAPINIC ACID	183.88	4.1	4.27	3.59	3.51	−1.75	I
8-IODOCATECHIN TETRAMETHYL ETHER	75.15	3.97	3.67	3.18	3.22	0.39	I
ABIETIC ACID	48.73	2.85	3.64	2.64	3.17	−1.78	I
ABSCISIC ACID (cis,trans; +/−)	0	2.9	3.1	2.41	2.88	−1.16	I
ACACETIN DIACETATE	85.86	3.6	3.54	3.23	3.18	−1.79	I
ACETOSYRINGONE	0	0	2.56	0	2.46	−1.22	I
ACONITIC ACID	−15.95	0	2.1	0	2.17	−4.29	I
AJMALINE	98.68	2.63	3.81	2.46	3.28	−0.5	I
ALBIZZIINE	−12.45	0	1.53	0	1.93	−4.53	I
alpha-MANGOSTIN	103.46	4	3.84	3.65	3.37	−1.43	I
alpha-TOXICAROL	150.65	3.78	3.82	3.36	3.35	−0.75	I
AMBELLINE	93.59	3.13	3.64	2.63	3.13	−1.55	I
ANDIROBIN	215.52	4.05	4.17	3.67	3.5	−2.19	I
ANDROGRAPHOLIDE	74.04	3.7	3.63	3.21	3.21	−1.19	I
ANGOLENSIC ACID. METHYL ESTER	187.06	4.01	4.26	3.62	3.5	−2.87	I
ANGOLENSIN (R)	29.1	2.97	3.07	2.63	2.96	−0.42	I
ANHYDROBRAZILIC ACID	19.01	2.4	2.81	1.8	2.72	0.14	I
ANTHOTHECOL	227.98	4.25	4.37	3.84	3.57	−2.86	I
ANTIAROL	0	0	2.48	0	2.32	−4.35	I
APHYLLIC ACID	41.17	2.74	3.16	2.46	2.95	−1.7	I
APIOLE	16.55	1.67	2.95	1.51	2.61	−5.98	I
ASIATIC ACID	182.51	3.99	4.38	3.73	3.62	−1.86	I
ATRANORIN	48.15	3.52	3.48	3.2	3.16	−1.7	I
AZADIRACHTIN	619.77	5.21	4.68	4.65	3.82	−3.47	I
BACCATIN III	278.44	4.9	4.44	4.38	3.66	−2.78	I
beta-AMYRIN	144.36	3.66	4.22	3.6	3.57	−1.91	I
beta-AMYRIN ACETATE	183.21	3.87	4.32	3.76	3.62	−2.07	I
beta-ESCIN	2640.9	5.39	4.92	4.91	4.3	−3.62	I
beta-MANGOSTIN	380.49	4.85	4.48	4.52	3.82	−0.71	I
beta-PELTATIN	162.63	4.19	3.88	3.55	3.31	−2.05	I
beta-TOXICAROL	152.26	3.79	3.8	3.41	3.35	−0.88	I
BETULINIC ACID	161.91	3.76	4.25	3.57	3.57	−1.63	I
BIOCHANIN A DIACETATE	85.5	3.64	3.56	3.26	3.18	−2.08	I
BIOCHANIN A. 7-METHYL ETHER	55.86	3.1	3.26	2.71	3.03	−0.79	I
BIOCHANIN A. DIMETHYL ETHER	60.82	3.19	3.37	2.84	3.05	−2.12	I
BISABOLOL ACETATE	0	2.81	3.14	2.71	2.91	−4.11	I
BUSSEIN	764.47	5.73	5	5.18	3.98	−4.95	I
BYSSOCHLAMIC ACID	56.95	3.46	3.71	2.65	3.12	−0.85	I
CAFESTOL ACETATE	121.68	3.39	3.79	3.03	3.32	−0.1	I
CARAPIN	192.89	4.08	4.23	3.66	3.51	−2.04	I
CARNOSINE	0	2.53	2.6	1.9	2.55	−2.47	I
CATECHIN PENTAACETATE	144.78	4.34	3.91	3.82	3.39	−1.55	I
CATECHIN TETRAMETHYLETHER	71.37	3.46	3.48	3.03	3.09	−2.49	I

Annex 1. *Cont.*

CEDRELONE	179.55	3.66	4.23	3.37	3.5	−2.09	I
CELLOBIOSE (D[+])	33.97	3.62	3.04	2.8	2.85	−0.98	I
CENTAUREIN	211.56	4.34	3.87	3.75	3.42	0.19	I
CEPHALOTAXINE	84.93	3.15	3.67	2.59	3.14	−1.17	I
CHELIDONINE (+)	128.03	3.47	3.75	3	3.26	−0.83	I
CHOLESTAN-3beta,5alpha,6beta-TRIOL	139.9	3.58	3.98	3.44	3.48	−0.62	I
CHOLESTERYL ACETATE	159.78	3.68	3.99	3.57	3.51	−0.69	I
CHONDROSINE	36.76	3.67	3.12	2.89	2.89	−1.38	I
CHRYSANTHEMIC ACID. ETHYL ESTER	0	1.39	2.68	1.39	2.59	−3.78	I
CHUKRASIN METHYL ETHER	715.41	5.61	5.02	5.1	3.95	−6.05	I
CINCHONIDINE	80.94	3.14	3.35	3.01	3.16	−0.72	I
CITROPTEN	13.76	1.54	2.7	1.11	2.58	−1.02	I
CITRULLINE	−18.67	2.21	1.89	1.4	2.18	−1.74	I
CLOVANEDIOL DIACETATE	55.96	3	3.63	2.53	3.12	−1.33	I
COLCHICEINE	75.92	3.89	3.87	3.35	3.23	−3.5	I
COLCHICINE	81.53	3.97	3.92	3.47	3.25	−4.28	I
COLFORSIN	71.76	3.69	4.11	3.33	3.3	−5.4	I
CRYPTOTANSHINONE	62.22	2.89	3.6	2.57	3.17	−0.57	I
D,L-threo-3-HYDROXYASPARTIC ACID	−11.07	0	1.46	0	1.93	−3.52	I
DAUNORUBICIN	250.86	4.46	4.08	3.98	3.57	0.26	I
DEACETYLGEDUNIN	189.32	3.82	4.23	3.46	3.5	−2.05	I
DECAHYDROGAMBOGIC ACID	488.85	4.92	4.5	4.62	3.87	−0.57	I
DEGUELIN(−)	143.41	3.67	3.78	3.31	3.33	−0.89	I
DEHYDRO (11,12)URSOLIC ACID LACTONE	184.12	3.79	4.35	3.65	3.61	−2.35	I
DELTALINE	181.15	3.39	4.4	2.92	3.46	−2.6	I
DEMETHYLNOBILETIN	84.49	3.62	3.67	3.2	3.15	−3.75	I
DEOXYGEDUNIN	184.29	4.23	4.3	3.86	3.53	−3.32	I
DEOXYGEDUNOL ACETATE	223.45	4.37	4.39	4	3.58	−3.49	I
DEOXYKHIVORIN	267.04	4.67	4.5	4.26	3.65	−3.95	I
DEOXYSAPPANONE B 7,3'-DIMETHYL ETHER ACETATE	86.97	3.41	3.44	3.03	3.16	−0.43	I
DERRUSTONE	90.86	3.35	3.4	2.9	3.09	−1.09	I
DIFFRACTAIC ACID	47.53	3.57	3.58	3.28	3.16	−3.28	I
DIFUCOL HEXAMETHYL ETHER	34.57	3.27	3.58	2.84	2.97	−6.34	I
DIHYDROCELASTRYL DIACETATE	247.18	4.31	4.39	4.03	3.68	−1.44	I
DIHYDROGAMBOGIC ACID	481.73	4.88	4.49	4.59	3.85	−0.95	I
DIHYDROGEDUNIC ACIDMETHYL ESTER	180.9	4.26	4.26	3.8	3.48	−3.65	I
DIHYDROJASMONIC ACID	0	2.43	2.71	2.09	2.7	−1.71	I
DIHYDROMYRISTICIN	13.24	1.47	2.63	1.26	2.54	−3	I
DIHYDROSAMIDIN	86.63	3.92	3.69	3.51	3.22	−3.2	I
DIPROTIN A	0	3.47	3.34	3.09	3.02	−2.91	I
DUARTIN (−)	65.61	3.28	3.44	2.91	3.07	−2.6	I
ECHINOCYSTIC ACID	170.52	3.93	4.32	3.72	3.61	−1.7	I
ENTANDROPHRAGMIN	898.5	5.6	4.88	5.09	3.99	−3.99	I
EPIAFZELECHIN TRIMETHYL ETHER	61.09	3.25	3.35	2.84	3.05	−1.54	I
EPOXYGEDUNIN	280.34	4.36	4.36	3.93	3.58	−3.01	I
ERGOSTEROL ACETATE	183.54	3.86	4.18	3.77	3.59	−1.3	I
ETHYL EVERNINATE	0	1.79	2.67	1.26	2.54	−1.63	I
EUGENITOL	12.56	0.69	2.7	0.69	2.66	0.22	I
EUGENYL BENZOATE	30.55	2.96	3.03	2.78	2.96	−1.28	I
EUPATORIN	69.85	3.43	3.45	2.97	3.09	−1.78	I
EUPATORIOCHROMENE	15.3	1.67	2.84	1.51	2.76	−0.34	I
EUPHOL ACETATE	176.8	3.86	4.22	3.77	3.59	−1.8	I
EUPHORBIASTEROID	298.53	4.56	4.28	4.2	3.65	−1.45	I
EVERNIC ACID	40.23	3.31	3.25	2.95	3.05	−1.03	I
EVERNINIC ACID	0	1.3	2.24	0.71	2.37	0.8	A
FISSINOLIDE	228.11	4.31	4.34	3.89	3.57	−2.78	I
FRAXIDIN METHYL ETHER	17.19	1.81	2.98	1.33	2.65	−2.95	I
FREQUENTIN	0	2.59	2.84	2.31	2.82	−0.69	I

Annex 1. *Cont.*

FRIEDELIN	142.51	3.63	4.24	3.58	3.57	−2.23	I
FUCOSTANOL	150.8	3.6	3.97	3.56	3.51	−0.64	I
FUMARPROTOCETRARIC ACID	125.97	4.18	3.85	3.7	3.36	−1.44	I
GAMBOGIC ACID AMIDE	98.04	3.89	3.79	3.54	3.36	−0.93	I
GAMBOGIC ACID AMIDE	443.87	4.8	4.43	4.51	3.83	−0.16	I
GANGALEOIDIN	70.07	3.77	3.73	3.19	3.2	−2.02	I
GANGLEOIDIN ACETATE	88.48	3.97	3.88	3.41	3.27	−2.73	I
GARDENIN B	73.39	3.43	3.56	3.05	3.11	−3.3	I
GEDUNOL	230.38	4.29	4.33	3.89	3.56	−3.14	I
GENKWANIN	50.06	3.08	3.15	2.71	3.01	−0.34	I
GERALDOL	54.15	3.3	3.23	2.77	3.03	0.19	I
GRISEOFULVIC ACID	52.2	3.15	3.55	2.59	3.06	−1.86	I
GRISEOFULVIN	56.93	3.39	3.65	2.83	3.08	−3.21	I
HEDERACOSIDE C	2844.2	5.44	4.91	4.95	4.32	−4.13	I
HEDERAGENIN	174.57	3.93	4.3	3.72	3.61	−1.52	I
HETEROPEUCENIN. METHYL ETHER	25.23	2.83	3.26	2.68	2.93	−4.61	I
HOMOPTEROCARPIN	64.45	2.91	3.25	2.55	3.03	−0.36	I
HYDROQUINIDINE	97.02	3.38	3.45	3.16	3.2	−0.95	I
HYDROXYPROGESTERONE	75.79	3.15	3.72	2.87	3.27	−0.37	I
HYMECROMONE METHYL ETHER	11.82	1.47	2.59	1.26	2.59	−1.33	I
HYPOXANTHINE	5.67	0	1.63	0	2.14	0.25	I
ICHTHYNONE	166.18	3.69	3.79	3.34	3.33	−1.23	I
INOSINE	42.33	2.91	2.92	2.11	2.73	−0.89	I
IRETOL	0	0	1.93	0	2.23	−0.57	I
ISOPIMPINELLIN	32.69	1.79	3.18	1.26	2.78	−1.03	I
ISOSAFROLE	9.9	1.2	2.26	0.92	2.47	0.88	A
ISOTECTORIGENIN TRIMETHYL ETHER	69.3	3.36	3.53	3	3.09	−3.36	I
ISOTECTORIGENIN. 7-METHYL ETHER	64.39	3.25	3.43	2.84	3.07	−1.98	I
KAINIC ACID	0	0	2.79	0	2.57	−0.82	I
KARANJIN	65.16	2.69	3.31	2.57	3.08	−1	I
KHAYANTHONE	322.96	4.67	4.51	4.26	3.67	−3.61	I
KHAYASIN C	243.02	4.37	4.35	3.96	3.6	−2.51	I
KHELLIN	35.79	1.61	3.3	1.61	2.84	−4.9	I
KHIVORIN	333.82	4.72	4.52	4.29	3.67	−3.87	I
KINETIN	36.46	2.72	2.55	2.3	2.65	−1.67	I
KUHLMANNIN	52.07	3.13	3.44	2.63	3.03	−2.04	I
LAGOCHILIN	61.47	3.36	3.83	2.84	3.21	−2.14	I
LANOSTEROL	146.22	3.66	4.11	3.61	3.54	−1.62	I
LAPACHOL	21.17	2.37	3.04	2.2	2.91	−0.88	I
LAPPACONITINE	416.24	4.44	4.48	4.08	3.7	−2.53	I
LARIXOL	30.12	3.01	3.61	2.84	3.13	−3.47	I
LARIXOL ACETATE	38.35	3.26	3.76	3.05	3.21	−3.53	I
LECANORIC ACID	36.97	3.21	3.17	2.9	3.03	−0.77	I
LEOIDIN DIMETHYL ETHER	78.86	3.99	3.91	3.44	3.26	−3.72	I
LIGUSTILIDE	13.92	1.67	2.57	1.51	2.67	0.06	I
LIMONIN	240.25	4.03	4.28	3.58	3.52	−2.12	I
LIQUIRITIGENIN DIMETHYL ETHER	52.47	3.06	3.16	2.75	3.01	−0.82	I
LOBARIC ACID	116.34	4.18	3.9	3.79	3.4	−1.58	I
LOGANIC ACID	79.08	3.65	3.32	3.15	3.09	−0.81	I
LOGANIN	85.57	3.75	3.39	3.24	3.11	−1.34	I
LONCHOCARPIC ACID	146.18	4.09	3.93	3.84	3.44	−2.02	I
MARMESIN ACETATE	52.7	3	3.23	2.55	2.98	−1.05	I
MELATONIN	22.9	2.65	2.79	2.21	2.76	−0.7	I
MELEZITOSE	132.93	4.44	3.58	3.65	3.24	0.07	I
MENTHYL BENZOATE	26.81	2.83	3	2.83	2.96	−2.1	I
METHYL COCLAURINE	58.31	3.15	3.29	2.87	3.08	−0.88	I
METHYLNORLICHEXANTHONE	39.75	2.64	3.23	2.21	2.95	−0.44	I
METHYLXANTHOXYLIN	0	1.2	2.83	0.92	2.54	−3.7	I
MUNDULONE	190.88	3.85	3.86	3.53	3.45	0.37	I

Annex 1. *Cont.*

MUNDULONE ACETATE	234.3	3.98	3.99	3.67	3.51	−0.17	I
NILOTICIN	213.75	3.87	4.15	3.7	3.58	−0.96	I
NOMILIN	254.75	4.31	4.42	3.85	3.57	−3.49	I
NONIC ACID	−19.75	1.99	2.31	1.4	2.38	−2.14	I
NORSTICTIC ACID	92.85	3.77	3.68	3.25	3.2	−2.11	I
OBLIQUIN	34.82	2.52	2.91	2.23	2.84	−0.71	I
ODORATONE	206.06	3.98	4.18	3.8	3.59	−1.16	I
OLEANOLIC ACID ACETATE	203.6	4.03	4.37	3.83	3.65	−1.87	I
ORSELLINIC ACID DIMETHYL ETHER	0	1.3	2.63	0.71	2.46	−1.24	I
ORSELLINIC ACID. ETHYL ESTER	0	1.2	2.52	0.92	2.51	−1.13	I
OSAJIN	134.28	3.83	3.8	3.61	3.41	−0.72	I
OSTHOL	21.9	2.62	3.06	2.49	2.85	−4.12	I
o-VERATRALDEHYDE	0	0	2.38	0	2.33	−3.05	I
OXONITINE	436.89	4.97	4.64	4.4	3.74	−3.4	I
PACHYRRHIZIN	120.84	3.46	3.46	3.03	3.18	0.06	I
PACHYRRHIZONE	143.53	3.55	3.67	3.09	3.26	−0.38	I
PACLITAXEL	963.42	5.33	4.73	4.87	4.06	−0.06	I
PAEONOL	0	1.2	2.21	0.92	2.38	−1.07	I
PENICILLIC ACID	0	0	2.31	0	2.28	−3.74	I
PERSITOL HEPTAACETATE	−86.01	4.77	3.78	4.06	3.16	−5.27	I
PEUCEDANIN	38.87	2.64	3.17	2.21	2.9	−1.34	I
PEUCENIN	23.32	2.37	3.09	2.2	2.91	−1.6	I
PHYSCION	42.59	2.62	3.39	2.26	3.03	−0.69	I
PIPLARTINE	40.16	3.26	3.23	2.83	2.97	−2.23	I
PODOFILOX	161.99	4.16	3.88	3.54	3.31	−2.13	I
PODOPHYLLOTOXIN ACETATE	196.18	4.37	4.01	3.75	3.38	−2.66	I
POMIFERIN	142.78	3.88	3.82	3.64	3.42	−0.53	I
PRIMULETIN	36.33	2.57	2.99	2.36	2.94	0.05	I
PRISTIMEROL	180.76	4	4.27	3.78	3.59	−1.79	I
PROTOPORPHYRIN IX	294.45	4.64	4.24	4.51	3.73	−0.92	I
PSEUDO-ANISATIN	36.73	0	3.61	0	3.01	1.79	A
PTERIN-6-CARBOXYLIC ACID	13.75	1.58	2.49	1	2.48	−0.14	I
PTERYXIN	84.81	4.02	3.69	3.64	3.22	−3.77	I
PYRROMYCIN	301.73	4.6	4.25	4.15	3.64	−0.54	I
QUASSIN	91.89	3.66	4.07	3.13	3.3	−3.44	I
QUEBRACHITOL	0	0	2.16	0	2.32	−0.62	I
QUERCETIN PENTAMETHYL ETHER	79.46	3.71	3.63	3.24	3.13	−3.61	I
RETUSIN 7-METHYL ETHER	55.86	3.1	3.27	2.71	3.03	−0.9	I
RHAMNETIN	57.76	3.39	3.28	2.83	3.05	0.17	I
ROBUSTONE	136.96	3.54	3.57	3.18	3.28	0.59	I
ROTENONE	148.91	3.67	3.78	3.31	3.33	−0.91	I
RUBESCENSIN A	93.33	2.91	3.97	2.53	3.3	−0.85	I
RUTILANTINONE	122.17	4	3.97	3.42	3.39	−0.52	I
SALICIN	27.45	3.18	2.89	2.67	2.84	−0.83	I
SALSOLIDINE	13.69	1.43	2.8	1.31	2.64	−2.85	I
SALSOLINE	11.67	0.69	2.64	0.69	2.61	−0.35	I
SALVINORIN A	134.4	4.13	4	3.65	3.35	−3.4	I
SALVINORIN B	108	3.7	3.89	3.23	3.28	−2.6	I
SAPPANONE A TRIMETHYL ETHER	73	3.33	3.34	2.95	3.1	−0.76	I
SCANDENIN	146.55	4.07	3.93	3.83	3.44	−2.05	I
SCANDENIN DIACETATE	208.19	4.4	4.14	4.13	3.55	−2.41	I
SELINIDIN	66.61	3.39	3.42	3.07	3.12	−1.71	I
SERICETIN	130.07	3.93	3.82	3.72	3.41	−1.36	I
SINAPIC ACID METHYL ETHER	0	2.71	2.88	1.89	2.61	−2.67	I
SINENSETIN	80.9	3.52	3.63	3.11	3.13	−3.51	I
SOLIDAGENONE	59.69	3.14	3.67	2.89	3.17	−2.99	I
STICTIC ACID	100.8	3.88	3.75	3.36	3.22	−2.93	I
STRYCHNINE	104.53	2.49	4.01	2.26	3.34	−0.31	I
SUMARESINOLIC ACID	170.79	3.91	4.33	3.71	3.61	−1.91	I
TANGERITIN	78.21	3.5	3.66	3.14	3.13	−4.32	I

Annex 1. *Cont.*

TETRAHYDROFURAN-3-YL)-6-OXO-10,10-DIMETHYLBICYCLO[7: 2: 0]UNDECANE 2-PROPYL-3-HYDROXY ETHYLENEPYRAN-4-ONE	0	0.85	2.51	0.41	2.47	0.53	I
TETRAHYDROPALMATINE	97	3.4	3.67	3.13	3.2	−3.04	I
THEOBROMINE	9.68	0	2.38	0	2.32	−3.39	I
TOTARALOLAL	47.04	2.79	3.73	2.57	3.17	−2.49	I
TOTAROL	42.4	2.62	3.69	2.49	3.16	−2.82	I
TOTAROL ACETATE METHYL ESTER	58.57	3.06	3.83	2.89	3.23	−3.51	I
TOTAROL-19-CARBOXYLIC ACID METHYL ESTER	57.08	3.04	3.83	2.78	3.21	−3.14	I
TRIACETYLRESVERATROL	53.36	3.41	3.36	3.1	3.14	−0.56	I
TRIDESACETOXYKHIVORIN	198.91	3.91	4.26	3.5	3.51	−2.05	I
TUBAIC ACID	16.45	1.73	2.8	1.39	2.73	0.54	I
URSINOIC ACID	25.49	2.87	3.23	2.41	2.9	−2.42	I
URSOLIC ACID	162.65	3.83	4.32	3.65	3.59	−1.99	I
UTILIN	839.81	5.5	4.87	4.98	3.96	−4.07	I
VERATRIC ACID	0	1.3	2.36	0.71	2.37	−0.61	I
VINCAMINE	102.14	3.23	3.98	2.98	3.28	−4.06	I
VINDOLINE	166.58	4.04	4.35	3.64	3.43	−5.81	I
VIOLASTYRENE	32.22	2.96	3.01	2.78	2.96	−1.11	I
VISNAGIN	28.98	1.39	3.06	1.39	2.78	−2.94	I
XANTHYLETIN	29.13	2.27	2.91	2.08	2.85	−0.6	I
XYLOCARPUS A	326.83	4.7	4.52	4.24	3.66	−3.8	I
ZEORIN	1646.1	5.45	4.95	4.96	4.24	-0.99	I
